# Whole-genome sequencing, as a powerful diagnostic tool in hearing loss, reveals novel variants in *PTPRQ* missed by whole-exome sequencing

**DOI:** 10.1186/s12920-025-02122-7

**Published:** 2025-03-31

**Authors:** Daniel Bengl, Asuman Koparir, Wahyu Eka Prastyo, Christian Remmele, Marcus Dittrich, Sophie Flandin, Waafa Shehata-Dieler, Clemens Grimm, Thomas Haaf, Michaela A. H. Hofrichter

**Affiliations:** 1 Institute of Human Genetics, Julius Maximilians University, Am Hubland, Würzburg, 97074 Bavaria Germany; 2 Center for Rare Diseases, University Clinics, Josef-Schneider-Straße 2, Würzburg, 97080 Bavaria Germany; 3 Bavarian Genomes Network for Rare Diseases, Technical University of Munich, Trogerstraße 32, Munich, 81675 Bavaria Germany; 4 Department of Bioinformatics, Julius Maximilians University, Am Hubland, Würzburg, 97074 Bavaria Germany; 5 Department of Otorhinolaryngology, Comprehensive Hearing Center, Würzburg University Hospital, Josef-Schneider-Straße 11, Würzburg, 97080 Bavaria Germany; 6 Chair of Biochemistry, Theodor-Boveri-Institute at the Biocentre University of Würzburg, Am Hubland, Würzburg, 97074 Bavaria Germany

**Keywords:** WGS, Hearing loss, PTPRQ, Alphafold, Deep intronic variant

## Abstract

**Background/objectives:**

Hearing loss (HL) is one of the most common congenital disorders, affecting 1-2 in 1,000 newborns. Modern genetic diagnostics using large gene panels and/or whole exome analysis (WES) can identify disease-causing mutations in 25-50 % of patients, with higher solve rates in individuals with earlier onset.

**Results:**

Here, we used whole-genome sequencing (WGS) to reanalyze 14 index patients/families who remained without genetic diagnosis by WES. We were able to identify the genetic cause of HL in 6 families ($\sim$43 %). Two families were diagnosed with DFNB84A caused by compound heterozygous recessive mutations in *PTPRQ*. Three of the four underlying variants, including a structural variant, a deep intronic variant, and a splice variant, escaped detection by WES. Minigene assays confirmed the pathogenicity of the intronic and the splice variants. In addition, we used protein 3D structure prediction and rigid ligand docking to study the pathogenicity of variants that escape nonsense-mediated decay.

**Conclusion:**

In our study, we present four novel variants in *PTPRQ*, three of which were detected only by WGS. To our knowledge, we report here the first pathogenic deep intronic *PTPRQ* variant causing HL. Our results suggest that the mutational spectrum of *PTPRQ* is not well covered by standard WES and that *PTPRQ*-associated hearing loss may be more frequent than previously thought. WGS provides an additional layer of information in the diagnostics of HL.

Introduction Hearing loss (HL) is the most common neurosensory disorders with 1–2 in 1,000 newborns and 3–4 in 1,000 adolescents being affected [1]. In developed countries approximately 80 % of congenital HL cases have a genetic basis [2]. Adequate hearing is crucial for the development of language and cognitive abilities. Individuals with congenital HL often exhibit challenges in both intellectual and social domains [3]. HL is characterized by enormous clinical and genetic diversity. Clinically, HL is classified into two main categories: syndromic (approximately 20–30 % of cases) and nonsyndromic (70 %−80 %). So far, more than 150 genes have been associated with nonsyndromic hearing loss (NSHL) and even many more with syndromic hearing loss (SHL). Molecular genetic testing has revealed pathogenic variants in *GJB2* in about 20 % of NSHL patients and in *STRC* in 5–10 % of cases, particularly in children exhibiting high-frequency HL [2].

Whole-exome sequencing (WES) has revealed disease-causing mutations in 20–30 % of the *GJB2*- and *STRC*-mutation-negative patients [4]. However, despite the enormous advancements in HL diagnostics, nearly half of NSHL cases remain undiagnosed by WES. Whole-genome sequencing (WGS) has emerged as the diagnostic tool of choice to study these unsolved cases. In addition to the mutations in protein-coding sequence detected by WES, it can resolve deep-intronic variants, structural variants (SVs), and variants in regulatory regions [5, 6].

The *PTPRQ* gene consists of 45 exons and is associated with an autosomal recessive (DFNB84A, MIM#613391) form of NSHL (Fig. 1A and B) [7]. *PTPRQ* encodes the tyrosine phosphatase receptor Q, a membrane protein required for the shaft connector and hair bundle formation in cochlea [8]. Expression of *PTPRQ* in the cochlea is primarily concentrated in the basal turn, the region responsible for detecting high-frequency sounds [8]. To date, more than 30 patients have been reported with DFNB84A. In addition, a single heterozygous nonsense variant (c.6881G>A) in the C-terminal region of *PTPRQ* in two different families has been reported to cause autosomal dominant NSHL (DFNA73, MIM#617663) [9, 10]. This substitution, c.6881G>A, truncates the protein at codon 2294, which is 6 amino acids from the end of the protein. The authors speculated that this variant may have a gain of function mechanism or a dominant negative effect.

**Fig. 1:**
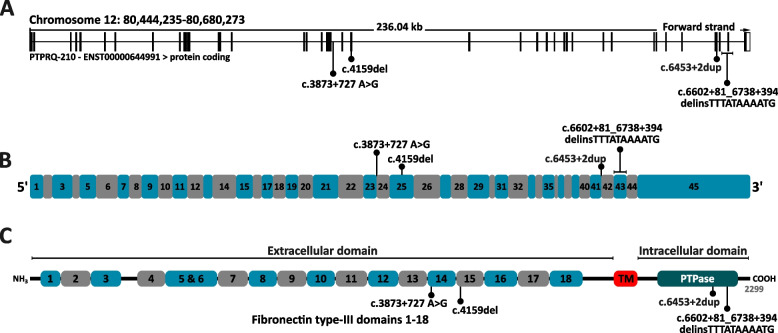
Structural overview of the human *PTPRQ* gene, exons, expressed protein and the location of identified variants. **A** Genomic structure and location of *PTPRQ* (adopted from [11]). **B** Exons constituting *PTPRQ*. **C** Structure and domains of the PTPRQ protein, retrieved from [12] (entry: Q9UMZ3). TM: Transmembrane domain; PTPase: tyrosine-protein phosphatase

In this study, we utilized WGS to re-analyze 14 unrelated index patients/families with NSHL, that remained unsolved in standard WES diagnostics. WGS identified the disease-causing variants in six (43 %) of 14 families. Interestingly, two independent families presented with compound heterozygous variants in *PTPRQ* (DFNB84A), three of which were not seen or misinterpreted in first-line WES diagnostics. The detected deep intronic and splice variant were analyzed by minigene assays and protein 3D structure prediction.

## Results

### Clinical description

**Fig. 2:**
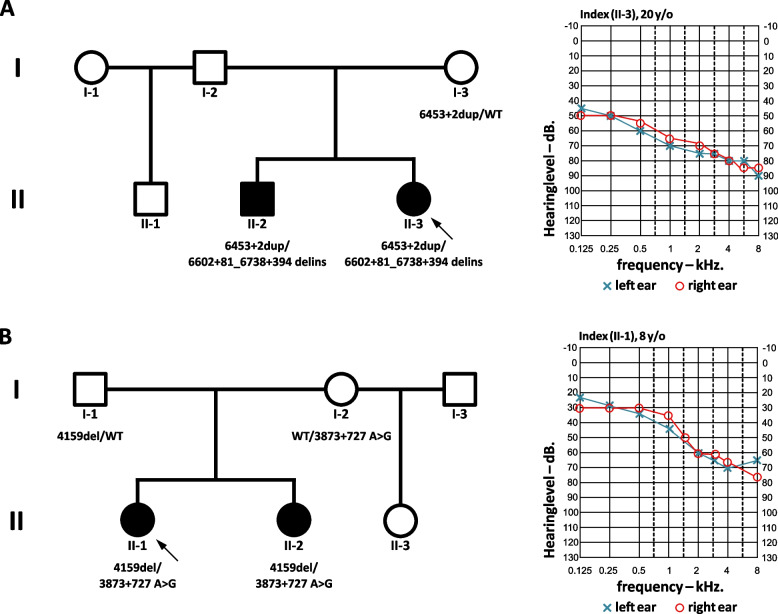
Pedigrees and audiograms of the index probands. Index probands are indicated by arrows. **A** Pedigree of family 1 with segregation of the *PTPRQ* variants c.6453+2dup and c.6602+81_6738+394delinsTTTATAAAATG. **B** Pedigree of family 2, segregating c.3873+727A>G and c.4159del. WT, wild type

In this study, we describe two German families. Family 1 consists of 6 members with two affected children suffering from bilateral NSHL. Three family members, the index patient II-3, her affected brother II-2, and the unaffected mother I-3, participated in the study (Fig. 2A). Both siblings failed the newborn hearing screening in both ears. The auditory brainstem responses (ABR) with click showed responses at 50 dB on both ears. Both siblings underwent regular audiological examinations, including pure tone audiogram and tympanogram. The index patient, now a 23-year-old woman, displayed a progressive moderate to severe HL of the mid- to high frequencies (Fig. 2A), which has been stable since the age of 11. Her brother showed a progressive severe HL of the mid- to high frequencies which has been stable since the age of 14. During the last examination, neither developmental delays nor vestibular dysfunction were observed in either sibling.

In family 2, the index patient II-1, her affected sister II-2, and the unaffected parents I-1 and I-2 could be analyzed (Fig. 2B). The index patient, now a 15-year-old girl, failed the newborn hearing screening in both ears. ABR showed responses at 60 dB on the left ear and 50 dB on the right ear. The index patient underwent regular audiological examinations, including pure tone audiogram and tympanogram, which indicated a mild to severe HL of the mid- to high frequencies (Fig. 2B). Neither developmental delays nor vestibular abnormalities were evident in her at the last examination. Her affected sister did not fail the newborn hearing screening, but audiological examinations presented similar results as the index patient. No vestibular dysfunction or motor delays were detected in her, but a sigmatism was noted at the age of four years. All affected individuals from both families use hearing aids and have good hearing outcomes. After clinical examination, additional symptoms and risk factors for HL, such as infections and trauma, were excluded.

### Molecular studies

In the first line, WES was performed for the index patients II-3 of family 1 and II-1 of family 2. On average 99 % of the target regions were covered (with at least 10 reads), and the average sequencing depth was 50-fold. In family 1, no pathogenic variants were detected. In family 2, WES revealed a novel heterozygous variant c.4159del, p.(Gln1387Lysfs*8) in exon 25 of *PTPRQ* (NM_001145026.2). Sanger sequencing confirmed the mutation in the index patient II-1, her affected sister II-2, and the normal-hearing father I-1 (Fig. 6A in Appendix). However, since the second mutation was missing, the case remained unsolved.

In the second line, WGS was performed. In family 1, the average sequencing depth was 40.75-fold and on average 91.73 % of the bases in the genome (hg38), 99.76 % of hearing loss genes (including introns) and 99.73 % of *PTPRQ* were covered with at least 10 reads (Table 3 in Appendix). The WGS analysis resulted in 4.86 million detected variants on average, filtered to 223 thousand rare variants, 1.05 thousand rare variants in hearing loss genes and 23 rare variants in *PTPRQ* (Table 2 in Appendix). Two variants in *PTPRQ* (NM_001145026.2) were identified in family 1 by WGS in the two affected siblings II-2 and II-3 (Fig. 5 in Appendix). The first variant was a duplication at the 5’ donor splice site of exon 41 at the c.6453+2 position (SpliceAI score for donor loss: 0.77), was misinterpreted as an artifact by WES. Due to the presence of an adenine homopolymer in this region, the sequencing platform generated errors, leading to the incorrect calling of three additional different variants, which complicated its evaluation. Additionally, the reads supporting the c.6453+2 duplication represented only 18.2% of all reads. The second variant was the c.6602+81_6738+394delinsTTTATAAAATG deletion-insertion variant spanning intron 42, exon 43 and intron 43 (no predicted influence on splicing). Both variants were validated by Sanger sequencing in the affected siblings (Fig. 5B and C in Appendix). The mother I-3 carried only the c.6453+2dup variant in a heterozygous state, consistent with compound heterozygosity in her affected children.

In family 2, WGS identified a novel heterozygous deep-intronic variant (SpliceAI score for donor gain: 0.94, for acceptor gain: 0.93), c.3873+727A>G,p.? in intron 23 of *PTPRQ* (NM_001145026.2), in addition to the already in WES identified heterozygous variant c.4159del, p.(Gln1387Lysfs*8) (Fig. 6C in Appendix). Sanger sequencing demonstrated heterozygosity for the deep intronic variant in the index patient II-1, her affected sister II-2, and her unaffected mother (Fig. 6B in Appendix), confirming the compound heterozygous state of the two variants in the affected siblings.

### Minigene assays of the *PTPRQ* c.6453+2dup and the deep-intronic variant

**Fig. 3:**
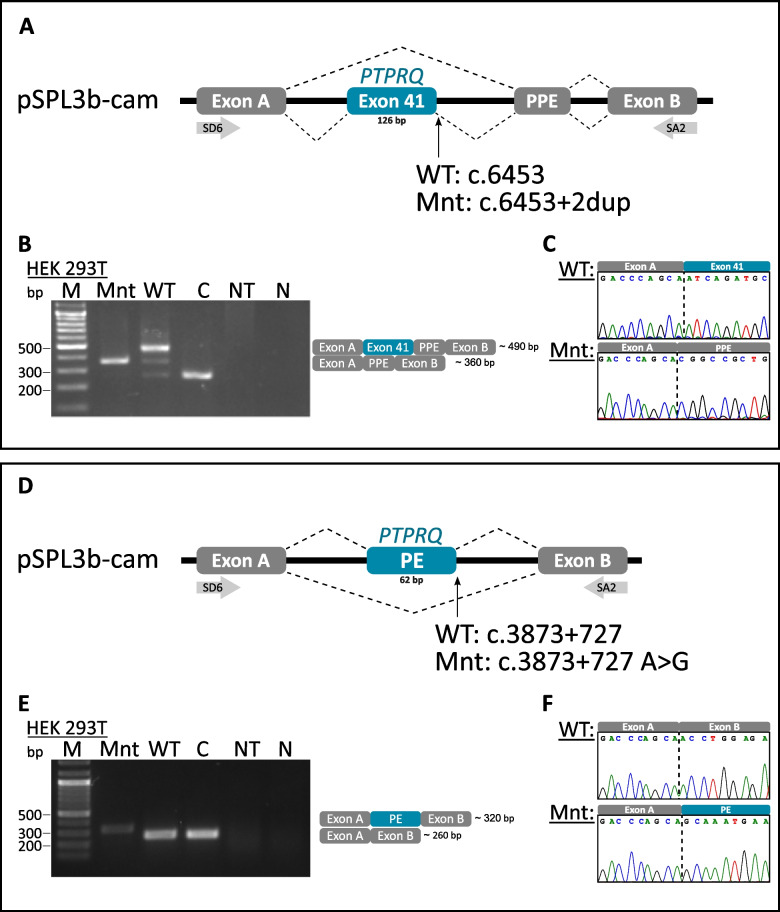
Splicing analysis of the c.6453+2dup and c.3873+727A>G variants. **A**, **D** Schematic illustrating the splicing behavior of the variant and wild type carrying construct. **B**, **E** Agarose gel electrophoresis of wild type and variant RT-PCR products. **C**, **F** Corresponding Sanger sequencing electropherograms of the products displayed in (**B** and **E**). WT: wild type; Mnt: mutant; C: control; NT: not transfected; N negative control; bp: basepairs; PE: pseudo exon; PPE: plasmid pseudo exon

To assess the functional consequences of the c.6453+2dup splice site variant, we conducted an in vitro minigene assay (Fig. 3A). Agarose gel electrophoresis revealed a size difference of 130 bp between the wild type (WT) (490 bp) and the mutant band (360 bp) (Fig. 3B). The control used the bare pSPL3b-cam plasmid, yielding a 260 bp band comprising artificial exons A and B. Sanger sequencing revealed the presence of exon 41 in the WT and its absence in the mutant transcript. In addition, a pseudoexon originating from the plasmid backbone (PPE) was identified in the mutant. The additional splice sites most likely arose during the cloning process. Thus, the mutant transcript consisted only of the artificial exons A, B and the PPE (Fig. 3C). Our minigene assay showed that the c.6453+2dup variant affects correct splicing of *PTPRQ* exon 41, resulting in an in-frame deletion in the tyrosine-protein phosphatase domain.

Similarly, we used a minigene assay to analyze the by SpliceAI predicted potential of the deep-intronic variant c.3873+727A>G to create a donor site and introduce an additional pseudo exon (Fig. 3D). To accomplish this, we used the intronic region surrounding the c.3873+727 position and the predicted complementary acceptor site as inserts for our plasmid constructs. After transfection, we found an elongated transcript from the mutant plasmid compared to the WT and control (Fig. 3E). Sanger sequencing confirmed the inclusion of a 62 bp pseudoexon (PE) which is not present in the WT (Fig. 3F). The WT plasmid, therefore, demonstrates splicing behavior closely resembling that of the control, as its insert is composed entirely of the wild-type intronic sequence. Our results suggest that during splicing of the *PTPRQ* pre-mRNA, the c.3873+727A>G variant functions as an additional donor site and activates an acceptor site located 62 bp upstream. This leads the inclusion of an extra exon after exon 23, causing a frameshift and a premature stop codon after 59 amino acids.

### Impact of *PTPRQ* variants on PTP-domain 3D structure

#### 3D modeling of PTPRQ and substrate interaction

Since the c.6453+2dup and the c.6602+81_6738+394delinsTTTATAAAATG variants do not cause premature stop codons, neither nonsense-mediated decay nor nonstop decay may account for their pathogenicity [13–16]. To visualize the impact on the affected phosphatase domain of PTPRQ, we used AlphaFold2 to predict the 3D structures of the active domains of the wild-type and variant proteins (Fig. 1C) [17].

**Fig. 4:**
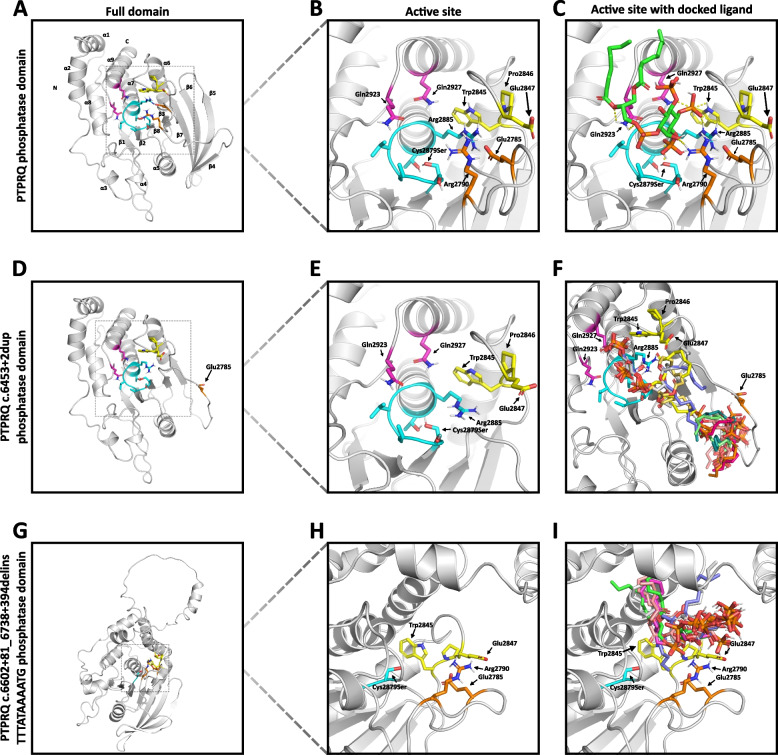
Ribbon representations of PTPRQ protein structure predictions using AlphaFold2 and rigid docking experiments. **A**, **D**, **G** PTPRQ wild type, c.6453+2dup and c.6602+81_6738+394delinsTTTATAAAATG phosphatase domains. **B**, **E**, **H** Close up of the active site of the PTPRQ phosphatase domains. **C**, **F**, **I** rigid docking results. For the wild type structure (**C**) the result with the highest affinity according to autodock vina was used to illustrate ligand binding. For the variants (**F** & **I**) docking results were superimposed. Distinct colors mark various segments of the active site: Cyan for the PTP-loop, magenta for the Q-loop, yellow for the WPE-loop, and orange for Arg2790 and Glu2785

**Fig. 5:**
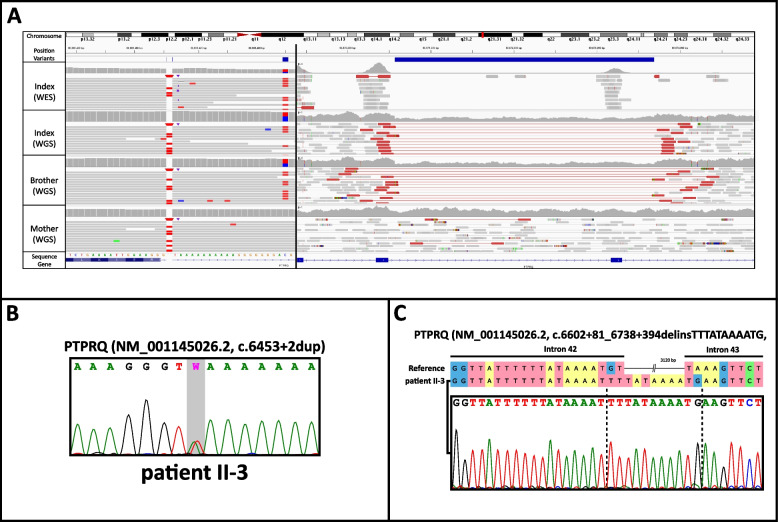
NGS and Sanger sequencing results of family 1. **A** Illustration of the detected c.6453+2dup and c.6602+81_6738+394delinsTTTATAAAATG variants of *PTPRQ* in IGV. Comparison of whole exome and whole genome data of the index patient, her affected brother and unaffected mother. **B** Validation of the c.6453+2dup variant by sanger sequencing (**C**) Validation of the breakpoint of the deletion and evidence of intronic insertion of a TTTATAAAATG stretch. In the second variant

**Fig. 6:**
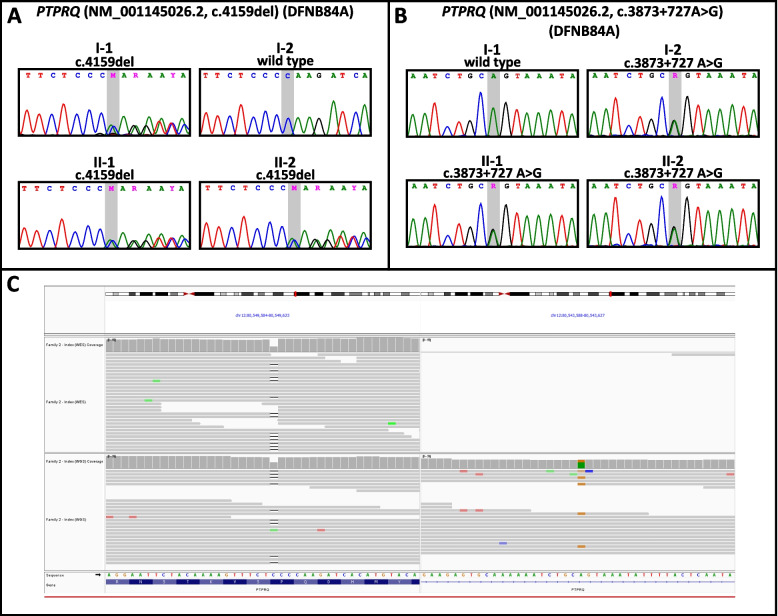
Segregation analysis of family 2. **A** Sanger sequencing results for all family members of the c.4159del variant. **B** Sanger sequencing results for all family members of the c.3873+727A>G variant. **C** Illustration of the detected c.4159del and c.3873+727A>G variants of *PTPRQ* in IGV. Comparison of whole exome and whole genome data of the index patient

The predicted WT structure with a detailed illustration of residues 2784 to 2790 is based on the published structure (PDB: 4IKC) with an RMSD of 0.631 [18]. The WT protein consists of eight twisted $\beta$-sheet strands in the center surrounded by $\alpha$1, $\alpha$2 and $\alpha$6-$\alpha$9 on the one side and $\alpha$3-$\alpha$5 on the other (Fig. 4A). The active site of PTPRQ adopts a typical conformation for a classical protein tyrosine phosphatase (PTP) (Fig. 4B), consisting of the phosphate-binding loop (PTP-loop) housing the catalytically important cysteine and conserved arginine residues, the WPE-loop and the Q-loop [18]. To explore potential interactions with one of the main substrates, phosphatidylinositol (3,4,5)-trisphosphate (PI(3,4,5)P_3_), we conducted rigid docking simulations using AutoDock Vina and the generated folding predictions (Fig. 4C) [18–20]. In our model the PI(3,4,5)P_3_ interacts with the catalytic Cys2879 via its phosphate residue at the 5’ position of the inositol group. The phosphate group at 4’ position forms hydrogenbonds with the sidechains of Arg2885 of the PTP-loop, Trp2845 of the WPE-loop and Gln2927 of the Q-loop. Additionally, Arg2790 forms a hydrogenbond with the hydroxy group at the 6’ position of the substrates inositol ring, while the Gln2923 interacts with the oxygen of the phosphoester bond. Our model highlights the significance of various conserved residues in the proper ligand binding of PTPRQ.

#### Effects of the splice variant on substrate interaction

The c.6453+2dup splice variant leads to the skipping of exons 41, thus removing 42 amino acids coding for the $\beta$-sheets $\beta$4-$\beta$6 (Fig. 4D). This deletion removes large portions of the so-called M6-loop, defined as the loop between $\beta$3 and $\beta$6 [18]. The AlphaFold2 prediction shows the remaining integrity of the C-terminal catalytic domain, featuring the PTP, WPE, and Q loops (Fig. 4E). The exon 41 skipping also removes the catalytically vital Arg2790 with the evolutionary conserved motif 6 (residues 2777–2781) remaining intact [21]. The Glu2785 residue is dislocated from the catalytic site, unable to interact with the substrate (Fig. 4D). These alterations appear to affect PI(3,4,5)P_3_ binding to the catalytic domain, with the substrate being docked reliably beneath the catalytic center, unable to interact with the WPE-, Q- or PTP-loop. We thus conclude that Arg2790 and the correct positioning of Glu2785 are crucial for proper ligand binding, underscoring the necessity of exons 41 for the overall activity of the phosphatase domain.

#### Influence of the c.6602+81_6738+394delinsTTTATAAAATG variant on substrate interaction

The c.6602+81_6738+394delinsTTTATAAAATG delins variant, which induces a frameshift, results in the translation of 111 amino acid residues before a premature stop codon is encountered. In the AlphaFold2 prediction, these amino acids form an outward-leaping loop atop the catalytic domain, with the central $\beta$-sheets flanked by $\alpha$-helices remaining intact (Fig. 4G). The deletion of exon 43 removes large parts of PTP-loop and the Q-loop, with the WPE and M6-loop remaining intact (Fig. 4H). In docking experiments, the PI(3,4,5)P_3_ substrate is displaced away from the remaining catalytic Cys2879Ser, with the inositol group facing outward of the binding pocket (Fig. 4I). Additionally, the outward-leaping loop seems to sterically obstruct the binding pocket, reducing its accessibility. This underscores the importance of the missing conserved amino acids for proper substrate binding and highlights the variant’s impact on steric properties.

### Variant interpretation

**Family 1:** The maternally inherited c.6453+2dup variant is reported three times in heterozygous but not in homozygous state in population database gnomAD v4.1.0, supporting PM2 evidence. The in vitro minigene assay revealed an impact on pre-mRNA splicing, justifying PS3 evidence. This variant results in an in-frame deletion including exon 41, which provides a PVS1_Strong. A report describing a patient with a deletion involving exons 40 and 41, underscoring the pathogenicity of our variant (ClinVar variation ID: SCV001762448.1) [22, 23].

The c.6602+81_6738+394delinsTTTATAAAATG deletion removes exon 43, which is located in the essential tyrosine phosphatase domain, and results in a frameshift with a stop codon after 111 amino acids, which is consistent with PVS1_strong evidence. The deletion cosegregates with the c.6453+2dup variant in the family, permitting evidence of PM3. Additionally, its absence from databases invokes PM2 evidence. Thus, the c.6453+2dup variant and the c.6602+81_6738+394delinsTTTATAAAATG deletion are classified as pathogenic and likely pathogenic according to the American College of Medical Genetics (ACMG) guidelines, respectively [24, 25].

**Family 2:** A novel heterozygous variant, c.4159del, p.(Gln1387Lysfs*8), is inherited from the unaffected father to his two affected daughters. It has not been documented in the literature or in population databases such as gnomAD v4.1.0, which provides PM2 evidence. The c.4159del variant induces a premature stop codon, resulting in a truncated protein with 1,394 amino acids, whereas the full-length protein has 2,299 amino acids. This fulfills the PVS1 criteria.

The deep intronic variant c.3873+727A>G is inherited from an unaffected mother. It has not been documented in population databases supporting PM2 evidence. Additionally, the splice assay results demonstrated an effect on pre-mRNA splicing, leading to a premature stop codon, providing support for PVS1 and PS3 evidence. Cosegregation with the p.(Gln1387Lysfs*8) variant within the family allows the application of the PM3 criteria. In summary, both variants in family 2 are pathogenic according to ACMG guidelines [24, 25].

## Discussion

In our study, we identified in two families diagnosed with high frequency HL four novel variants in the *PTPRQ*, three of which were detected only by WGS due to PCR artifacts and insufficient coverage by WES. These drawbacks of WES, particularly the lack of coverage of regulatory regions and uneven sequencing depths in GC-rich regions, are well known [26–28]. Furthermore, WES is known to have difficulty identifying variants in homopolymeric regions, and detecting INDELs in these areas is particularly challenging [29].

To date, the Deafness Variation Database (DVD) annotates 39 *PTPRQ* variants as likely pathogenic or pathogenic [30, 31]. In family 1, we identified c.6453+2dup and c.6602+81_6738+394delinsTTTATAAAATG variants. Similar to our cases, some of the reported variants affect crucial segments of the phosphatase domain. For instance, the previously reported c.6475C>T variant causes a premature stop codon upstream of the WPE loop, removing the PTP- and Q-loop [32].

Another variant, c.6739-1G>A, alters the splice acceptor site of exon 44, causing exon skipping and a frameshift, removing parts of the Q-loop including Gln2927 [33]. Collectively, these variants argue in favor of the functional importance of the affected PTPRQ domains.

The results of our minigene assays support the hypothesis that the c.6453+2dup variant induces aberrant splicing and skipping of exon 41 (Fig. 3), which ultimately results in the removal of Arg2790 and the displacement of the residue Glu2785 from the catalytic site. Kinetic experiments [18] demonstrated the importance of these residues in PTPRQ’s pNPP and PI(3,4,5)P_3_ dephosphorylation activity. This aligns with our docking analysis showing that the substrate is not efficiently docked in the binding pocket of the mutant (c.6453+2dup) protein. Our results also support the proposed interaction of Arg2790 with the substrate (Fig. 4C and F) [18].

The c.6602+81_6738+394delinsTTTATAAAATG variant causes a frameshift, resulting in a protein with 36 additional amino acids, extending beyond the canonical stop codon of the wild-type protein. These form a loop in the alphafold prediction, extending across the catalytic domain (Fig. 4G). The deletion of exon 43 removes large parts of the PTP-loop, including Arg2885, which is functionally important for proteins of the PTP family. It also removes the Q-loop, which is crucial for phosphoester hydrolysis and substrate specificity (Fig. 4H) [34–37].

PTPRQ was initially identified as a protein tyrosine phosphatase; however, subsequent analyses revealed that it has relatively low PTPase activity. Instead, PTPRQ functions primarily as a phosphatidylinositol phosphatase (PIPase) and is capable of dephosphorylating phosphatidylinositol 4,5-bisphosphate (PI(4,5)P_2_) [38–40]. PI(4,5)P_2_ is a crucial component in hair cells, where it plays essential roles in mechanotransduction and both fast and slow adaptation. In these cells, PI(4,5)P_2_ is localized in the distal part of the hair bundle but is absent at the apical surface and in the taper region [41]. This spatial distribution is inversely related to that of PTPRQ, which localizes mainly at the base of the hair bundle, with its concentration diminishing toward the apex [41]. This reciprocal distribution suggests that PTPRQ is essential for maintaining a PIP_2_-free zone at the base of the stereocilia. This is speculated to be important to regulate the activity of cytoskeletal-associated proteins such as RDX or CLIC5, which are also located in this region and required for stereocilia integrity [42–45].

Our variants, c.6453+2dup and c.6602+81_6738+394delinsTTTATAAAATG, both affect the phosphatase domain of PTPRQ. These mutations likely reduce its activity toward PI(4,5)P_2_, potentially leading to an accumulation of PI(4,5)P_2_ at the base of the hair bundle and thus affecting stereocilia formation.

In family 2, we identified the c.4159del and c.3873+727A>G variants. The c.3873+727A>G variant is notable as it is the first deep-intronic variant reported in *PTPRQ*, leading to the inclusion of a PE. Both variants cause a frameshift closer to the N-terminal end, deleting the phosphatase domain, several fibronectin domains and the transmembrane motive. This is expected to cause nonsense-mediated decay. In addition to ours, several other pathogenic variants, i.e. c.3717 C>A and c.4370del variants [46], have been reported in this region, affecting the PTPRQ protein in similar ways.

*PTPRQ* spans 45 exons and encodes a protein consisting of 2,299 amino acids. Considering its large size, relatively few mutations in this gene have been reported so far. Here 2 (14 %) of 14 NSHL patients/families, who remained unsolved by WES, were diagnosed by WGS with DFNB84A, due to compound heterozygous mutations in *PTPRQ*. Three of the 4 pathogenic mutations escaped detection by WES because they were not or only insufficiently covered. *PTPRQ* is among the genes with large sequence gaps in the WES datasets. In contrast, the average coverage of *PTPRQ* including intronic regions was 99.73 % with at least 10 reads in our WGS datasets (Table 3 in Appendix). Thus, WGS allows for the identification and diagnostic assessment of intronic variants.

Collectively our data suggest that DFNB84A may be more frequent than previously thought. However, due to the small sample size (*N* = 14) this finding should be interpreted with caution. Future studies with larger cohorts are required to obtain a more robust understanding and confirm our findings. In our experience with >250 HI patients/families, WES leads to a genetic diagnosis in 25–35 % of *GJB2*- and *STRC*-mutation-negative cases, with higher diagnostic rates achieved for individuals with an early (congenital or infant) onset of HL. Thus, a substantial proportion of patients remains currently unsolved. Switching from WES to WGS will definitely improve the diagnostic rate, and *PTPRQ* is likely among the top candidate genes, which will benefit from WGS analysis, following the exclusion of disease-causing mutations in *GJB2* and *STRC*.

## Material and methods

### Whole exome sequencing

Exome capture was performed according to the Illumina Nextera Rapid Capture Enrichment library preparation protocol (Illumina, San Diego, California, USA) using 50 ng of genomic DNA. Paired-end sequencing of the libraries was performed with a NextSeq500 sequencer and a v2 reagent kit (Illumina). The sequences were mapped to the human genome reference (NCBI build37/hg19 version) using the Burrows-Wheeler Aligner. Variants were called and analyzed using GensearchNGS software (PhenoSystems SA, Braine le Chateau, Belgium). Variants with a coverage of <10, a Phred-scaled quality of <15, a frequency of <15, and an MAF of > 2 % were neglected. Six control samples from healthy individuals were used for filtering out platform artifacts. Alamut Visual software (Interactive Biosoftware, Rouen, France), including prediction tools like SIFT, MutationTaster, and PolyPhen-2, was used for variant prioritization. Potential effects of a variant on pre-mRNA splicing were evaluated by SpliceSiteFinder-like, MaxEntScan, NNSPLICE, and GeneSplicer. Population databases like gnomAD revealed the population frequency of a given variant in a heterozygous or homozygous state.

### Whole genome sequencing

Library preparation was performed with the Illumina DNA PCR-Free Library Preparation kit (Illumina) using >300 ng of genomic DNA input. Paired-end sequencing was performed on an Illumina NovaSeq 6000TM and the v1.5 reagent kit (Illumina). Sequences were mapped with using bwa mem 0.7.17-r1188 [47] to the human reference sequence GRCh38 [48]. SNVs and INDELs were identified with deepvariant 1.6.1 [49] and SVs (deletions, duplications, insertions) with manta 1.6.0 [50] respectively. Variants were called, filtered by FAF <= 2 % and analyzed using gnomAD 4.1 [51], and the variant effects predicted by Ensembl VEP 112 Gencode v46 basic [52] as well as potential splice-altering variant effects by SpliceAI 1.3.1 (masked, D=500) [53]. Genes associated with hearing loss were obtained from Deafness Variation Database v9 comprising of 224 genes [31]. Read coverage was calculated with mosdepth 0.3.8 [54] with the following parameters –fast-mode -F 3844 -Q 5.

### Sanger sequencing

Sanger sequencing was used for validation of variants identified by NGS and for segregation analysis. PCR amplification was done using the Platinum Taq DNA-Polymerase (Invitrogen, Carlsbad, USA) with standard cycling conditions. Primers (Table 1 in Appendix) were designed using Primer3 [55]. Sequencing was performed with an ABI 3130xl 16-capillary sequencer (Life Technologies, Carlsbad, CA, USA). Data were analyzed with Gensearch 4.3 software (PhenoSystems SA, Wallonia, Belgium).

### Minigene assay

For variants predicted by SpliceAI to affect splicing (here *PTPRQ* c.6453+2dup and c.3873+727A>G), the in vitro minigene assay was used to validate their effects. The method was employed as previously described [56]. Briefly, wild-type and mutant *PTPRQ* exon 41, or the pseudoexon region, were PCR-amplified from the proband’s and a control individual’s DNA using primers with XhoI and NotI restriction sites (Table 1 in Appendix). The PCR products contained the exon along with approximately 150 bp of flanking upstream and downstream sequences (400 bp for the pseudoexon). After amplification, the products were purified, digested with restriction enzymes, and cloned into the linearized pSPL3b-cam exon-trapping vector. The resulting constructs were introduced into DH5$\alpha$ competent cells (NEB 5-alpha, New England Biolabs, Germany), purified via mini-prep, and the sequences of both wild-type and mutant vectors were confirmed by Sanger sequencing.

HEK 293T cells (ATCC) were transfected with 2 $\mu$g of the respective pSPL3b-cam vector (wild-type or mutant) at a density of $2.25\times 10^{5}$ cells/mL, using 6 $\mu$L of FuGENE®HD Transfection Reagent (Promega, Madison, WI, USA). Controls included the empty pSPL3b-cam vector and negative transfection reactions. After 24 hours, transfected cells were harvested for RNA extraction via phenol-chloroform. Approximately 1 $\mu$g of RNA was reverse transcribed into cDNA using the FIREScript®RT cDNA synthesis kit (Soli BioDyne, Estonia), following the manufacturer’s protocol. The cDNA was PCR-amplified using vector-specific primers SD6 and SA2 (Table 1 in Appendix). PCR products were visualized on a 1 % agarose gel and confirmed by Sanger sequencing.

### Structure prediction and molecular docking

Alphafold2 was used for the creation of models of the WT PTPRQ phosphatase domain, and the identified variants affecting this domain [17]. Bioinformatics software used in this study include auto dock vina software [19, 20], PyMOL (Schrödinger: The PyMOL Molecular Graphics System, Version 2.6.0a0, unpublished), ApE [57], AutoDock Tools suite [58] and online resources such as the National Center for Biotechnology Information [59]. The PDBQT files of the receptor and ligand were parameterized using the AutoDock Tools suite. This involved the addition of polar hydrogen atoms, assignment of Kollman Charges, and definition of torsional degrees of freedom for the ligand. The receptor grid was also defined using AutoDockTools and was set to include all relevant known residues for ligand binding. The center was set to be in the proximity of the Cys2879 residue. Spacing of the grid points was set to 0.375 Å.

After preparation of the ligand and receptor rigid docking experiments were conducted using the auto dock vina software. Exhaustiveness was set to 8. Further configurations were kept to their default values. The results were then loaded into pymol for visualization.

## Data Availability

All datasets supporting the findings of this study are available within the paper and its additional files.

## Appendix A Supplements

**Table 1:** Primer used in this study. Overhangs with respective restriction sites are highlighted by the use of (*)

Primer	Sequence
***PTPRQ Ex41 For***	ACAATGACGCTTATGACTGATGA
***PTPRQ Ex41 Rev***	CAGGAGATGAGTGACTTGG
***PTPRQ delins Ex43 For***	TGCCTTCAACCTTCATTGTGG
***PTPRQ delins Ex43 REV***	TTATGGTTTTACTGGCCCTGC
***PTPRQ MG int23 fw XhoI***	*TCATACTCGAG*ATGGAAAACACAAGCAAGCG
***PTPRQ MG int23 rev NotI***	*ATCATTGCGGCCGC*ACAACCACACTCTAACACACG
***PTPRQ MG int23 seq***	CAGAAAGAAAGTCATTGTTCCCG
***PTPRQ MG XhoI fw***	*TCATACTCGAG*TTCATCTAATACTGTGAGTCATC
***PTPRQ MG NotI rev***	*ATCATTGCGGCCGC*TGTTCAGAAGACAATACAAAAGCA
***pSPL3b cam MG seq***	ATATCTGGGATCCTGCAGCG
***PTPRQ intron 23-F***	AGCAGCACCAAGGGAATCTT
***PTPRQ intron 23-R***	ACCGCTATGCTAAGAGAGCC
**SD6**	TCTGAGTCACCTGGACAACC
**SA2**	ATCTCAGTGGTATTTGTGAGC

**Table 2:** Overview of all detected as well as filtered variants (SNVs & INDELs) of whole genome data of the index patient II-3 (family 1), the brother (II-2) and mother (I-3) as well as the index patient II-1 (family 2). Filter conditions consist of a FAF95 (gnomAD 4.1.0) $\le$ 2 %, in hearing loss genes (Deafness Variation Database) and the *PTPRQ* gene

Proband	Detected variants (SNVs & INDELs)	Rare (FAF95 $\le$ 2 %)	In HL genes (DVD)	In *PTPRQ* gene
Family 1 Index (II-3)	4,883,405	226,010	1,044	25
Family 1 Brother (II-2)	4,828,616	216,310	1,007	21
Family 1 Mother (I-3)	4,867,654	225,579	1,096	23
Family 2 Index (II-1)	4,833,828	208,122	1,020	13

**Table 3:** Overview of the read coverage for the genome (hg38), hearing loss genes in the Deafness Variation Database including introns (224 genes with 23,441,668 bp) and *PTPRQ* including introns (278,096 bp)

Proband	Ø read depth	Bp covered >= 10 reads	Bp in HL genes covered > 10 reads	Bp in *PTPRQ* covered >10 reads
Family 1 Index (II-3)	40.76	91.57 %	99.76 %	99.73 %
Family 1 Brother (II-2)	41.24	92.08 %	99.75 %	99.70 %
Family 1 Mother (I-3)	40.25	91.53 %	99.77 %	99.77 %
Family 2 Index (II-1)	19.11	90.00 %	98.55 %	98.23 %
